# Horizontal jumping biomechanics among elite female handball players with and without anterior cruciate ligament reconstruction: an ISU based study

**DOI:** 10.1186/s13102-019-0142-8

**Published:** 2019-11-18

**Authors:** Igor Setuain, Eder Bikandi, Francisco Antonio Amú Ruiz, Fernando Urtasun, Mikel Izquierdo

**Affiliations:** 1 Department of Health Sciences, Public University of Navarra, Campus of Tudela, Av. de Tarazona s/n, 31500 Tudela, Navarra Spain; 2 Clinical Research Department, TDN. Advanced Rehabilitation Center, Pamplona, Spain; 3 Athletic Club Football team. Medical Service, Bilbao, Spain; 4 Department of Physical Education and Sports, Universidad del Valle, Cali, Colombia

**Keywords:** Knee, ACL injury, Functional evaluation, Inertial sensor, Biomechanics

## Abstract

**Background:**

Handball is a strenuous body-contact team sport that places high loads on the knee joint. Anterior cruciate ligament (ACL) tear is one of the most devastating injuries that any handball player can suffer, and female athletes are at particular risk due to their intrinsic anatomical, hormonal, neuromuscular and biomechanical characteristics. The purpose of this study was to analyze the horizontal jumping biomechanics of female elite handball players with or without previous ACL reconstruction.

**Methods:**

Twenty-one female participants (6 with previous ACL reconstruction and 15 uninjured controls) were recruited. Two horizontal hopping tasks were evaluated using inertial sensor unit (ISU)-based technology to assess jumping biomechanics through a direct mechanics-based approach.

**Results:**

The athletes with previous ACL reconstruction demonstrated a significant (*P* < 0.05) reduction in the unilateral triple hop for distance compared with the healthy controls. Furthermore, during the initial propulsive phase of the unilateral cross-over hop, the control participants generated significantly (*P* < 0.05) higher force values in the mediolateral direction (the X axis) with their dominant limb compared with the ACL-reconstructed (ACL-R) limb of previously injured participants.

**Conclusions:**

Three-dimensional horizontal jumping biomechanics analyses using ISU-based technologies could provide clinicians with more accurate information regarding the horizontal jumping biomechanical patterns among elite handball female athletes. Furthermore, several mechanical alterations could still be observed among those players who had undergone previous ACL reconstruction, even when several years have passed since the original ACL injury.

## Background

Handball is a body-contact team sport that elicits high-intensity maneuvers such as abrupt changes in direction, velocity and sudden single leg landings [1, 2]. The nature of the sport and the high intensity of games, makes the knee joint to be exposed to many stressful forces that could result the anterior cruciate ligament (ACL), rupture, which constitutes one of the most devastating injuries among handball players [3, 4].

The reported incidences of ACL injury for male and female handball athletes are approximately 0.24 and 0.86 injuries per 1000 h of exposure, respectively [5]. Therefore, female athletes are 6 to 10 times more likely to suffer an ACL injury than their male counterparts during the same jumping and pivoting tasks [5, 6]. Anatomical, hormonal and neuromuscular differences between sexes have been proposed as explanatory factors for this discrepancy in the ACL injury rates between sexes [7–10].

The clinical relevance of ACL injury does not rest solely on the ligament disruption itself; the functional implications of concomitant associated knee injuries for the athletes’ function can play an important role in the clinical prognosis of the athlete after injury [11]. Additionally, the scientific literature lacks information regarding the best clinical practices for rehabilitation programs or universal functional and clinical evaluation criteria for resuming the sport after injury [11].

This ambiguity may expose the athlete to both higher risk of graft rupture and a new injury of the healthy contralateral knee [12]. Thus, the detection and monitoring of subjects with a higher risk of injury or re-injury using functional, biomechanical or neuromuscular screening evaluations appears to be crucial either for prevention and rehabilitation in sports medicine [13].

Functional performance evaluations have traditionally been highlighted as a key point in relation to decisions regarding resuming play after ACL injury [2, 13–16]. In this context, unilateral hopping tests have demonstrated a good ability to identify lower limb impairments during both vertical and horizontal jumping maneuvers [15–17].

Several biomechanical and neuromuscular impairments at the trunk, hip and knee joint levels have been widely reported in the literature as a result of motion analysis and inverse mechanics procedures during the abovementioned and other sport-specific tasks [18–21]. Unfortunately, these testing procedures require from expensive and complex laboratory resources (such as camera-motion analysis systems and/or force plates) and are associated with a high financial investment and trained staffs that are familiar with such laboratory-derived procedures. The recent development of ISU-based biomechanical evaluations presents clinicians with the opportunity to perform several functional and biomechanical jumping evaluations on the training court itself [22–28].

In relation to handball, Myklebust et al. [29] observed long-term differences in strength, jumping test scores and anterior-posterior knee joint laxity between ACL-injured and uninjured professional and recreational players after an injury. In addition, Setuain et al. [28] presented a validation study that reported promising results validating the utilization of the ISU versus force plate recordings during vertical jumping tasks. Later, the same research group probed the potential of ISU-based evaluations to assess vertical jumping biomechanical among both female [25] and male [26] elite handball players in relation to previous ACL injury. The authors found long-term, sex-specific functional adaptations after ACL reconstruction, being thefemale athletes more likely than males to experience lasting biomechanical jumping alterations after an ACL reconstruction [25]. The application of the ISU-based biomechanical jumping to identify movement pattern alterations after ACL injury has also been proven in previous studies [22, 23].

The aim of this study was to examine the biomechanical differences in horizontal jumping between elite female handball players with previous ACL reconstruction who had returned to their previous sport activity, and level-, sex-, and age-matched pairs of control counterparts. The hypothesis of the present research was that the ACL-R players would present lasting biomechanical alterations in terms of greater supported three-axis peak forces during single-limb horizontal jumping maneuvers compared with their control counterparts, despite have continued with elite competition for several years after the original ACL injury.

## Methods

A descriptive case series study design was selected. The examinations were conducted at the athlete’s habitual training court. The jumping task battery included the unilateral triple hop for distance (UTHD) and the unilateral triple cross-over hop for distance (COHD). These tests have been established as reliable methods for evaluating lower limb function in relation to ACL injury in previous investigations [16, 30, 31].

### Subjects

Twenty-one female elite handball players competing in their highest national division league and European championships were recruited. The sample comprised 6 athletes who had undergone ACL reconstruction, two of them bilaterally (age 26.4 ± 1.4 years; height 169.0 ± 1.6 cm; and weight 61.8 ± 1.4 kg), and 15 uninjured controls (age 25.1 ± 1.4 years; height 175.0 ± 1.4 cm; and weight 69.5 ± 1.8 kg). Among the athletes with bilateral reconstructions, both limbs were recorded as ACL-R limbs. The average and standard deviation of the data collection time since surgical reconstruction was 6.0 ± 3.5 years. For the control group, athletes who had sustained a previous lower limb injury lasting more than 6 weeks were excluded to avoid jumping pattern bias due to potential functional alterations resulting from severe lower extremity injury. The participants and coaches were informed in detail about the experimental procedures and the possible risks and benefits of the project, which was approved by the Ethical Committee of the Public University of Navarra and performed according to the Declaration of Helsinki.

### Equipment

An inertial orientation tracker (MTx, 3DOF Human Orientation Tracker, Xsens Technologies B.V. Enschede, The Netherlands) was attached over the L3-L4 region of the subject’s lumbar spine and provided data on kinematic and kinetic variables such as accelerations, orientations and velocity at a sampling rate of 100 Hz. A technical explanation describing the inertial sensor-derived variables has been previously provided (Additional file 1: Appendix A) [32]. Furthermore, a 10-m-long measuring tape was utilized to measure the distance in each horizontal jumping task. The last heel contact was recorded for the final measure.

### Procedures

Lower limb dominance was determined as previously described by Bencke et al. [2] in their work with handball players. The limb that pushed off the ground for a jump when a regular handball throw was performed was considered dominant [2]. All the participants performed the test at the beginning of a routine training session conducted during the competitive season and at least 48 h after their last competition. The jumping methodology used in this trial has been published previously [17, 24, 30, 31, 33–36]. The subjects were instructed that during the execution of each maneuver, they should keep their hands on their hips. No added technical instructions about the jumping modality were given to the athletes to avoid modifications during the task performance. The participants started in a single-limb stance position. They then performed three consecutive horizontal hops as far as possible, holding the position for at least 1 second after the last landing. For the COHD, the subjects adopted the same starting position and executed three consecutive cross-over hops outside two lanes separated by a 15-cm-wide tape attached on the floor, trying to land as far as possible while maintaining their balance for 1 second at the final landing. The first jumping step was interiorly directed. A practice trial was performed to ensure the participant’s comfort and safety and was followed by two further test trials interspersed with 30 s of rest. The jumping tasks were performed in order from easiest to most complex to avoid possible injury risks associated with the intensity of the maneuver. The participants thus started with the UTHD and ended with the COHD.

**Fig. 1:**
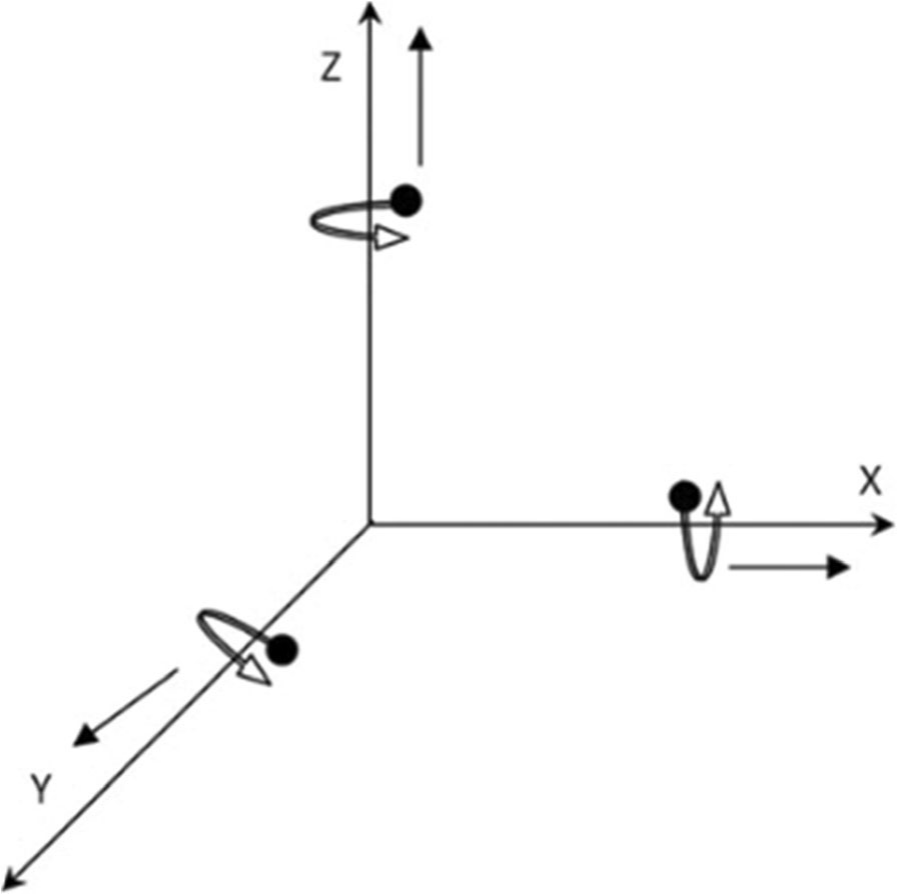
Z-axis (vertical), X-axis (medial-lateral) and Y-axis (anterior-posterior) orientations

ISU provides linear acceleration values in a sensor-fixed Cartesian reference frame (XYZ). Before starting the measurement, the inertial sensor unit is calibrated and the sensor axes are aligned with anatomical directions. The acceleration signal consists of gravitational and inertial components. The inertial sensor unit registers gravity as a static vertical component, in addition to the dynamic acceleration caused by changes in velocity during locomotion. The gravity component must be subtracted to estimate the dynamic acceleration. The 3D orientation data provide the position of the inertial unit with respect to the gravitational vector, allowing the calculation of the inertial component for each axis. The gravitational constant was estimated by leaving the inertial sensor unit still on a flat surface for 2 seconds. In previous studies [25–28], body-worn inertial sensor and accompanying custom algorithms has demonstrated high agreement and reliability levels compared with force plates, [28] (Fig. 1).

### Data processing and analysis

The data reported by the sensor was analyzed using direct mechanics-based procedures that considered the subject as a mechanical system and estimated the movement and actuation of forces through the center of mass displacement [37–39]. As previously mentioned, the human center of gravity is considered to be located at the L3 lumbar spine level, where the ISU was placed. The data processing description was previously published by this research group [29].

**Fig. 2:**
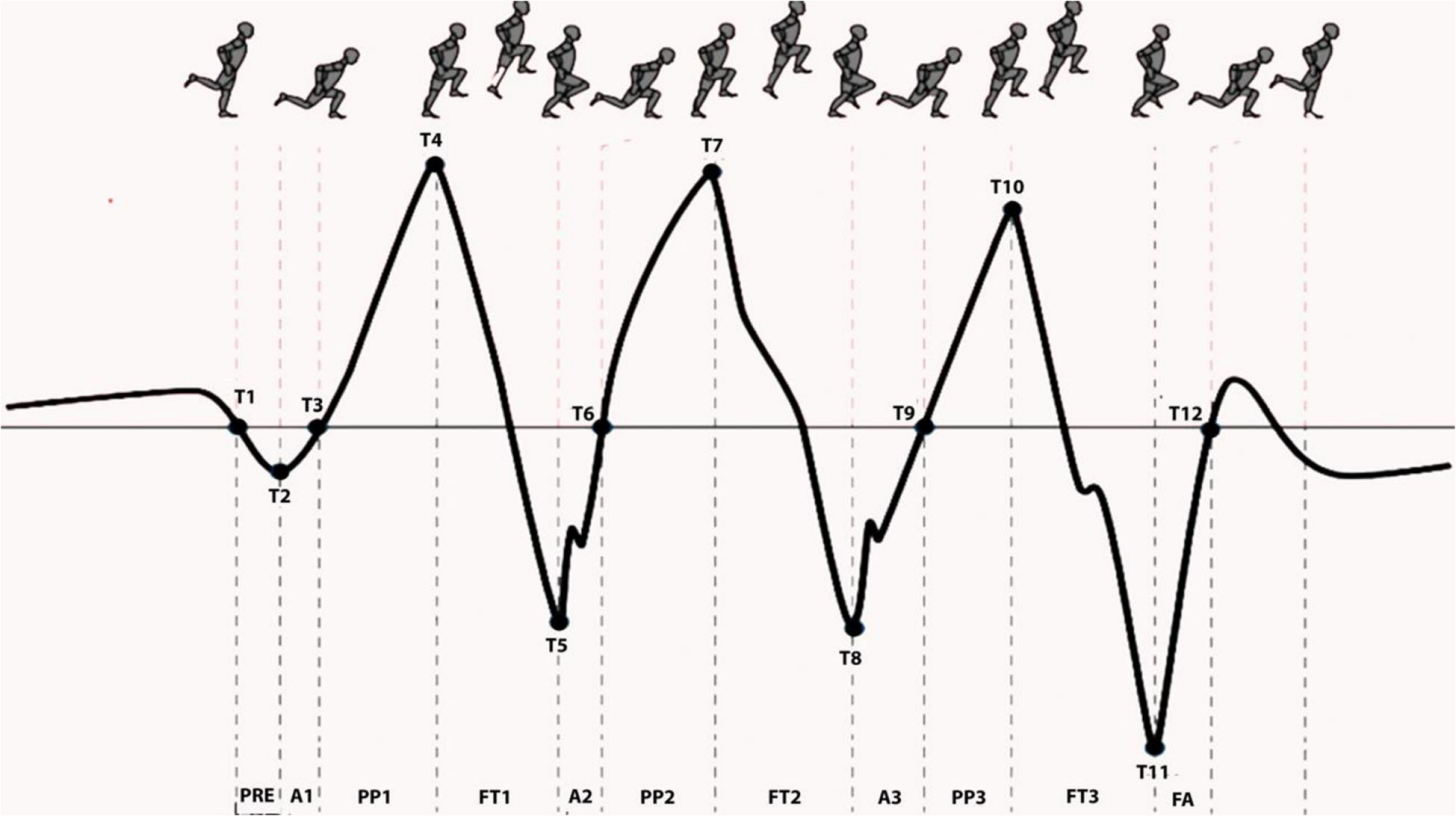
Horizontal jumping task jumping phases by velocity by time curve analysis description The segment T1-T3 represents the negative passive and active work (pre-stretch) corresponding to the propulsive phase (PP). The T3 event corresponded to the instant the *Z*-velocity first passed zero (when the centre of mass of the athlete was in its lowest position) during the transition between the initial absorption (A1) or pre-load and the propulsive phase (PP1) of the jump. The PP1 concluded in T4, when the maximal vertical velocity (propulsive phase) was achieved. Therefore, the segment T2-T3 represents the countermovement of the jump, and consequently, the segment T3-T4 corresponds to the PP1. Segment T4-t5 represents the flight time of the first jump (FT1). The same curve cut-off points were described thorough the whole triple hop analysed. Thus, absorptive (eccentric, T5-T6; T8-T9 and T11-T12) and propulsive (concentric; T6-T7, T9-T10) phases were similarly described

Briefly, in order to facilitate the biomechanical analysis of the jump, the task was divided into separate phases. The identified phases were based on the results obtained from the vertical velocity curve recordings (Z-axis) through a self-customized computer application implemented with MatLab 7.11 (MathWorksInc; Natick, MA, USA). The *Z*- velocity signal was used to distinguish the boundaries between the different phases of both tasks and were considered positive when the subject moved upwards (corresponding to the propulsive phases of the three consecutive jumps) and negative when subject moved downwards (corresponding to the pre-loading and landing phases). The different phases of the jumping task have been described succinctly in previous studies [25–29] (Fig. 2).

Lastly, the mechanical efficiency ratio (ME) calculation was defined as the ratio between the jumping performance (cm) and the sum of the peak ground reaction forces supported at the centre of mass level (N). The amount of the sum of three-dimensional forces would penalize or benefit the ratio in the horizontal jumping task. The ME, aims to determine to what extent the supported peak ground reaction forces are during the absorptive phases, in relation to the distance reached during the maneuver. Supporting greater peak ground reaction forces during the absorptive phases, could lead to a more harmful mechanical overload which could increase the injury risk.

$$ \boldsymbol{ME}=\frac{\boldsymbol{performance}\ \left(\boldsymbol{cm}\right)}{\left(\boldsymbol{Fx}+\boldsymbol{Fy}+\boldsymbol{Fz}\right)} $$

### Statistical analysis

Descriptive statistics (mean, standard error of the mean and IC values at 95%) were calculated for all the collected variables (weight in kg; height in cm; performance in cm; 3 axis GRFs in N).

Afterwards, descriptive statistics for the selected variable groups (ACL-R injured limb, ACL-R healthy limb, Control dominant limb) were applied. After normal distribution of the data and variances equality were checked through the Shapiro-Wilk and Levene tests respectively, a 2 X 2 (group by limb) multivariate analysis of variance (ANOVA) was performed to analyse interaction levels between factors. The dominant limb of the control group was matched to the involved limb of the ACL-R group and the non-dominant limb was matched to the non-involved limb of the ACLR group [19]. Thus, if between groups interaction was observed a one-way analysis of variance was performed in order to detect with subsequent Bonferroni post hoc comparisons, the existing differences between limb us with only one fixed factor (group; ACL-R vs controls). When the variance equality was rejected, the Tamhane’s post hoc test was performed. The significance level was set at *p* < 0.05. “SPSS® statistical software (V. 20.0, Chicago, IL, USA) was used for the abovementioned statistical calculations.

Apart from that, intra and inter-group differences were analysed using magnitude-based inferences (MBI). This statistical method was chosen in order to highlight the practical significance over the statistical (*p* value) significance, emphasizing that the magnitude of an effect would be more relevant than any statistically significant effect especially in the clinical practice or when treating elite athlete’s data [40, 41]. The magnitudes of the smallest worthwhile differences were identified by the determination of the effect sizes (Cohen’s d) for between-limbs and between group comparisons, using means and standard deviations for each group of variables. Values for Cohen’s d statistics were interpreted as follows: < 0.15 for trivial, 0.15 to 0.4 for small, 0.4 to 0.75 for medium, 0.75 to 1.10 for large and > 1.10 for very large differences [41].

## Results

After the data processing, the number of analysed limbs in both control and ACL-R group was the following: 8 ACL-R reconstructed limbs and 4 ACL-R in both UTHD and COHD maneuvers; 13 dominant and non-dominant limbs in the UTHD and 14 dominant and non-dominant limbs in the COHD of the control group. The ACL-R players were significantly (*p* < 0.05) lighter and smaller than their non-ACL-R counterparts. No significant interaction effects were found between factors for UTHD and COHD tests. Therefore, the results are delimitated to the description of the main effects observed.

### Unilateral triple hop for distance (UTHD)

**Fig. 3:**
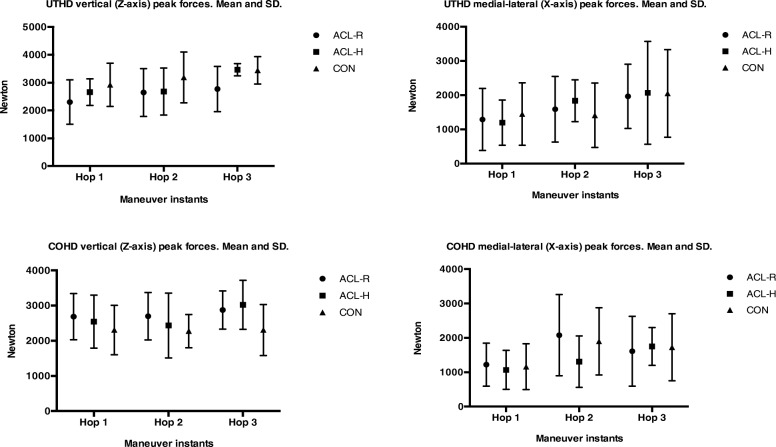
Between groups peak vertical and medial-lateral forces comparison during the unilateral triple hop for distance (UTHD) and the unilateral cross over hop for distance maneuvers. Mean and SD. Abbreviations: (UTHD), unilateral triple hop for distance; (COHD), unilateral cross over hop for distance; ACL-R, anterior cruciate ligament group-reconstructed limb; ACL-H, anterior cruciate ligament group-healthy limb; control group-dominant limb

Regarding the UTHD, the dominant limb of the controls reached a significantly better distance performance on the UTHD task compared with the injured limb of the ACL-R participants (*p* < 0.05). Indeed a non statistical trend although a large effect size was find in realtion to a gretater X mediolateral force production during the first hop in controls in comparison to ACL- reconstructed players. (Table 1). No further significant differences were found for any time or force variables (Fig. 3).

**Table 1:** Horizontal jumping performance for unilateral triple hop and unilateral cross-over hop for distance. Descriptive statistics, significance and effect size calculations for each group

		ACLR Injured Limb	ACLR Healthy Limb	Control Dominant Limb	Control Non-Dominant Limb	Significance (*p*)	*ES (d)*
UTHD	n	8	4	15	15		
	Performance	389 ± 61.05	398.25 ± 87.76	436 ± 37.84	430.29 ± 47.91	0.047*	*d = 0.925^*
	95% CI	337.97–440.03	258.61–537.89	411.95–460.05	402.62–457.95		
UCOHD	n	8	4	15	15		
	Performance	289.63 ± 58.24	310.5 ± 70.90	326.14 ± 44.84	329.31 ± 60.61	0.115	*d = 0.7025*
	95% CI	240.94–338.31	197.68–423.32	300.25–352.03	292.68–365.94		

Values are mean ± standard deviation, 95% confidence interval (inferior – superior value). *P* value from ANOVA calculations between ACLR injured limb and Control Dominant Limb. Standardised effect size interpreted as Cohen’s d values between ACLR injured limb and Control Dominant Limb. Abbreviations: *UTHD* Unilateral triple hop for distance, *UCOHD* Unilateral cross-over hop for distance, *n* Sample size, *SD* Standard deviation, *95% CI* 95% confidence interval, *ES* Effect size, *d* Cohen’s *d*. * = *p* < .05. ^ = *d* > 0.8

The ACL-R limbs of cases demonstrated a trend towards greater mechanical efficiency ratios (0.079 ± 0.02 vs. 0.070 ± 0.05; Cohen’s d = 0.4) when executing this horizontally oriented jumping task (Table 2).

**Table 2:** Horizontal jumping performance and three-dimensional force-based mechanical efficiency ratios. Descriptive statistics and effect size (Cohen’s *d*) calculations

Horizontal Jumping Tasks	ACLR Injured limb			ACLR Healthy Limb			Control Dominant Limb			*ACLR Injured* vs *ACLR Healthy*		*ACLR Injured* vs *Control Dom*	
	*n*	Mean (±SD)	95% CI	*n*	Mean (±SD)	95% CI	*n*	Mean (±SD)	95% CI	*ES (d)*	*Difference*	*ES (d)*	*Difference*
*d* > 1.10	8	0,079 (±0,022)	0,061 - 0,097	4	0,072 (±0,014)	0,049 - 0,094	13	0,070 (±0,021)	0,057 - 0,083	*0,379*	*small*	*0,418*	*medium*
*d* > 1.10	8	0,058 (±0,015)	0,046 - 0,071	4	0,064 (±0,022)	0,0287 - 0,1001	14	0,085 (±0,023)	0,072 - 0,098	*0,318*	*small*	*1,39*	*very large* ^a^

Values are mean ± standard deviation, 95% confidence interval and standardised effect size. Abbreviations: UTHD, unilateral triple hop for distance; UCOHD, unilateral cross over hop for distance; n, sample size; SD, standard deviation; 95% CI, 95% confidence interval; ES, effect size; *d*, Cohen’s *d*. ^a^ *d* > 1.10

### Triple cross-over hop for distance (COHD)

Regarding the COHD, no significant differences were found between the groups in terms of performance (reached distance) (Table 1). However, a significant group-by-limb interaction was observed for the PP X-axis forces (F = 4.353; *p* = 0.010). The Bonferroni post-hoc analysis revealed that the dominant limbs of the controls displayed significantly greater X-medial-lateral axis forces than the injured limbs of the ACL-R group (*p* < 0.05). No significant differences were found for the remaining analyzed variables (Fig. 3).

The ACL-R limbs of the cases demonstrated a trend towards lower mechanical efficiency ratios (0.058 ± 0.02 vs. 0.085 ± 0.02; Cohen’s d = 1.4) when executing this side-to-side and horizontally oriented jumping task.

For more information, available complementary material is included about the 3-axial forces results for the UTHD (Additional file 1: Appendix B) and the COHD (Additional file 1: Appendix C).

## Discussion

The purpose of this study was to examine the biomechanics of two horizontal hopping tasks among top-level professional female handball athletes using an ISU-based methodology. The analysis focused on the identification of persistent jumping biomechanics adaptations in the ACL-R limbs of previously injured athletes. The results of the present work showed that although the ACL-R participants had returned to full competition at high intensity and exigency levels, slight jumping biomechanics alterations seemed to persist.

Consequently, the previously ACL-injured limbs of the cases showed lower UTHD performance in terms of distance (Table 1), and reduced mediolateral force generation on the propulsive phases of this horizontally oriented jumps, specially in the COHD maneuver (Fig. 3). These findings may suggest that at the initial propulsion (the pre-loading phase preceding the first hop), the ACL-R limbs of the previously injured athletes generated lower frontal plane forces compared with the dominant limbs of the control athletes. Furthermore, during the execution of both horizontal jumping tasks, the ACL-R athletes were more prone (although not significantly) to generate lower Z-axis (vertical) and Y-axis (horizontal) forces. Interestingly, the newly proposed mechanical efficiency ratios demonstrated a trend towards lower values for the ACL-R limbs of the cases compared with the dominant limbs of the controls when executing this horizontally oriented jumping maneuvers, specially the CTHD. This could highlight that female handball players exhibit greater peak external force penalization (supporting ground reaction forces) when jumping with their previously ACL-R limb for the distance reached in comparison to that supported by controls. These results partially agree the study hypothesis, which posited that the ACL-R players would experience lasting biomechanical movement pattern alterations in terms of greater supporting three-axis peak forces during single-limb horizontal jumping maneuvers compared with their control counterparts despite having performed in elite competition for several years since the original ACL injury.

This results, contrast with those obtained by the same research group employing the same jumping test battery and biomechanical analysis methodology among male elite handball players. In that study, the authors did not find any meaningful biomechanical adaptations among previoulsy ACL reconstructed in comparison to control (non ACL injured) players. In this sense, it seems that male handball professional players are able to recover their lower limb full performance capacities without lasting biomechanical alterations that can be in contrast observed among their female counterparts. Although evidence exists referring no sex influence in relation to increased risk for ACL graft failure among sportspeople, [42] may be, this statistical trend would change when controlling for sex, handball sport, and level of competence of the participants. This question should be addressed in properly designed investigations.

Traditionally, lower limb functional evaluations have been carried out in order to determine the athlete capacities with regard to return to sport participation. tIndeed, jumping biomechanical have also been performed in relation to injury risk factor identification showing huge correlation between poor unilateral limb performance values and knee dynamic instability [31]. In this context, ground reaction forces acting at the trunk level have been considered to have significant effects on lower limb segment behavior due to the inertia moment of force generation [43, 44]. Consequently, frontal plane kinematic or kinetic parameters measured at the trunk level have been shown to be significantly associated with knee valgus production [43]. In this context, ISU systems have become a reliable instrumentation for trunk displacement-derived 3D force calculations in different functional tasks [25–28]. It has been shown, an upward trunk position when landing from a jump could lead to greater anterior shear forces at the knee joint and higher vertical peak ground reaction forces, exposing the ACL to a higher injury risk [35, 43].

Thus, despite knee joint moment description is not possible when analysing a jumping task through a direct mechanics approach, by placing an ISU on the L3-L4 level, clinicians by using this jumping biomechanical analysis method, could look for jumping aberrant patterns identification that have been previously linked to a greater knee joint injury risk due to excessive mechanical overload during high demand athletic tasks.

In this way, it is possible that ACL-R female athletes, could have developed lasting movement pattern adaptations during single-limb actions in the attempt to improve lower limb stiffness through movement pattern reprogramming at the central nervous system level [45, 46]. This fact would help to explain the smaller medial-lateral force produced at the center of mass level during both UTHD and COHD task, as a positive effect of the rehabilitation.. These results are in contrast with previous investigations from the same research group and cohort of athletes that analyzed vertical jumping maneuvers [26]. In that research, ACL-reconstructed athletes generated higher medial-laterally oriented peak forces than their control counterparts. In the authors’ opinion, this controversy could arise from a specific jumping direction-based motor retraining strategy adopted among cases to preserve knee joint integrity. In fact, the reduced mechanical efficiency ratios observed for the ACLR limbs of cases on the COHD task, which is known to place higher valgus stress on the knee joint than the UTHD, could support this hypothesis. However, this assumption should be adequately tested with studies designed to answer the specific question.

The identification of lasting functional and biomechanical jumping alterations several years after the injury in both the present and previous research [25–27], could be linked to an inadequate rehabilitation process or the approval of excessively early return to play by sports medicine staff when managing ACL injuries. This fact becomes clinically relevant in this context, as the time lapse between the time of reconstruction and retourn to sport participation, is know to affet ACL graft failure [42], The application of the ISU bed biomechanical jumping evaluations, could become useful for a more accurate motion analysis at the clinical setting level that would allow the clinician to plan an objective, clinically reasonable rehabilitation program based on the observed biomechanical alterations.

Some potential limitations could be observed in the present study. Given the uniqueness of the analyzed population, which was limited to an exclusive cohort of female professional handball athletes, the results should be interpreted with caution and in relation to this sport level, discipline and sex. Additionally, there was a lack of standardization of the postoperative rehabilitation protocols and the graft type used for the ligament repair among the ACL-R athletes. The heterogeneity of the rehabilitation process may have biased the long-term outcome in terms of physical activity level and sport-specific performance. However, previous studies have reported that no differences exist between reconstructions using different graft types in relation to long-term function of the knee [29]. Furthermore, the use of a single ISU placed at the trunk level limited the information collected regarding the knee joint biomechanics.. The, net moments of force calculations for specific joints were outside the scope of the present study which in turn tries to describe the centre of mass behavior thorough a direct mechanics approach. This is not as exhaustive as inverse mechanics procedures, but instead could be more friendly (in the field testing) and easy to handle for sport clinicians.

## Conclusions

In conclusion, elite female handball players with previous ACL reconstruction demonstrated a attenuated jumping capacity in the THD test. Indeed, they also displayed lower X-medial-lateral axis peak force generation, especially during the first propulsive phase of the CTHD. This fact could be interpreted as a protective effect against the lower limb collapse. As main clinical implication, ISU systems can aid the implementation of real-time simple biomechanical jumping examinations by sports medicine professionals in clinical settings to reduce the residual uncertainty that often arises during the ACL rehabilitation process regarding the return to sports. However, due to the uniqueness of the analyzed cohort the present results must be considered with caution and restricted to the intrinsic characteristics of these top level female handball players.

## Supplementary information

**Additional file 1: Appendix A.** Technical explanation of ISU technology-derived analysis [32]. **Appendix B.** UTHD manoeuvre phases breakdown in terms of centre of mass force orientation values. Descriptive statistics and effect size calculations. **Appendix C.** UCOHD manoeuvre phases breakdown in terms of centre of mass force orientation values. Descriptive statistics and effect size calculations.

## Data Availability

The datasets used and/or analysed during the current study are available from the corresponding author on reasonable request.

## Abbreviations

ACL: Anterior Cruciate Ligament
ACL-R: ACL-Reconstructed
COHD: Cross-Over Hop for Distance
GRF: Ground Reaction Force
ISU: Inertial Sensor Unit
ME: Mechanical Efficiency ratio
UTHD: Unilateral Triple Hop for Distance
