# Supplementary material for: Horizontal jumping biomechanics among elite female handball players with and without anterior cruciate ligament reconstruction: an ISU based study
Source: BMC Sports Sci Med Rehabil. 2019 Nov 18;11:30. doi: 10.1186/s13102-019-0142-8 (PMC6859617; doi:10.1186/s13102-019-0142-8)
Supplement: Supplementary file 1 — Additional file 1: Appendix A. Technical explanation of ISU technology-derived analysis [32]. Appendix B. UTHD manoeuvre phases breakdown in terms of centre of mass force orientation values. Descriptive statistics and effect size calculations. Appendix C. UCOHD manoeuvre phases breakdown in terms of centre of mass force orientation values. Descriptive statistics and effect size calculations. [file 13102_2019_142_MOESM1_ESM.docx]

Appendix A. Technical explanation of ISU technology-derived analysis [32].

*Instrumentation*

An ISU integrating 3 accelerometers, 3 gyroscopes and 3 magnetometers (MTx, Xsens Technologies B.V. Enschede, Netherlands) attached over the L3 region of the subject’s lumbar spine provided the kinematic data recorded in each trial at a sampling rate of 100 Hz.MTx combines itself nine individual MEMS sensors to furnish accurate3D orientation as well as other kinematic data such as: 3D acceleration, 3D rate of turn (rate gyro) and 3D earth-magnetic field.

Optical motion analysis system (Vicon Nexus 1.0) was used as truth-reference and it was time synchronized with the MTx to compare both signal results.


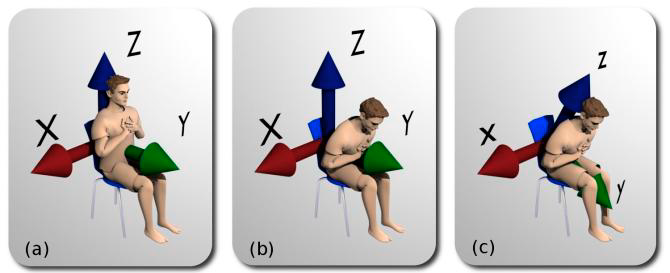
The ISU provided linear acceleration and rate of turn in a sensor-fixed Cartesian reference frame (x-y-z). Before the beginning of the test, with the subject sitting on the chair and his back in upright position, the sensor-fixed reference frame was aligned with the Earth-fixed global reference frame (XYZ), whose Z axis lies on the vertical pointing upwards, its X axis lies on the lateral direction and its Y axis on the anterior-posterior direction (Figure A1).

Figure A1. Changes in global and IU's local Cartesian reference axes when the subject is trying to stand up at the beginning of the 30-s CST. The first figure, (a), depicts the initial position; global and local reference axes coincide. When the subject changes position, the global axis remains unchanged (b) whereas the IU's local reference axis rotates with the physical device (c).

Orientation data consisting in the Euler angles (in XYZ or roll-pitch-yaw order) defined the rotation that aligned the global axis to the sensor-fixed reference frame at each time instant. Then, linear acceleration in the global reference frame was obtained from the acceleration and orientation data provided by the IU (Figure A2A). Furthermore, optical data were also collected using a 100Hz six-camera Vicon system (Vicon Motion System, Oxford, UK), in order to check the new method's accuracy. Specifically, in our study, a Vicon Nexus 1.0 was employed, using only three from the six available cameras. They were previously calibrated and the data from the two systems were time-synchronized through sync pulses in order to compare both of them in an off-line analysis with Matlab (Math Works, Massachusetts, USA).One 4 mm Vicon reflective marker was placed on the MTx to acquire its three-dimensional position for subsequent comparisons.


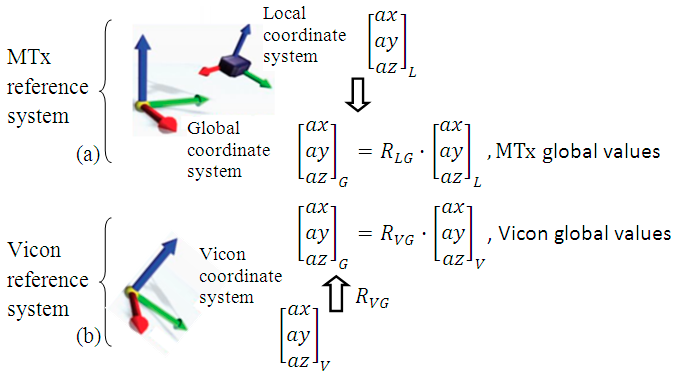


Figure A2: Reference systems changes to obtain the global values from MTx and Vicon. Sub-indexes “L”, “G” and “V” refer to the MTx local, global and Vicon local coordinate systems respectively and RLG and RVG to the rotation matrices to change coordinates from the first indicated reference system to the second one.

*Signal Processing*

*Drift effect correction.*


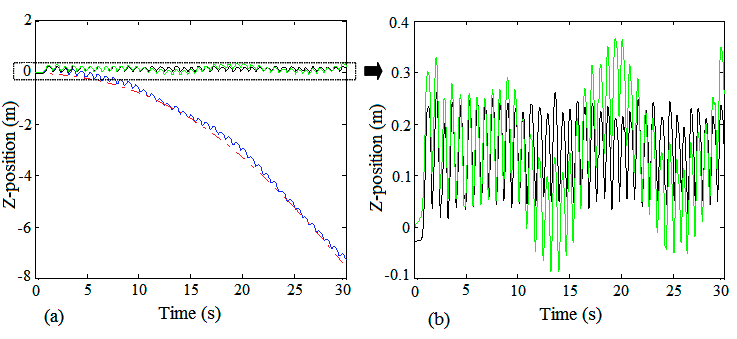
Z-position signal, obtained through double integration of the Z-acceleration, was used to detect the subject’s up and down positions and hence automatically obtain the number of complete sit-stand-sit repetitions during the 30-s CST. However, the raw Z-acceleration signal provided by the ISU has to be treated as previously mentioned. Firstly, the coordinate reference system needed to be changed from local to global. Secondly, the gravity acceleration component, roughly estimated as 9.8 m/s2, had to be removed(Figure A3).

Figure A3.(a) shows the Z-position signal (blue line) gravity error correction (green line), and the Vicon reference signal (black line). Red line is the tendency line based on fourth level polynomial estimation that tracks the gravity error, Part (b) shows the corrected and reference signal enlargement.

Finally, relative position was obtained through double integration of the acceleration data (Figure A4), assuming resting initial conditions. However, this straightforward process was hindered by noise in the acceleration signal as well as by approximation errors due to numerical integration. This drift effect that occurs for various reasons (e.g., vibration or environmental temperature fluctuations) can, in practice, make the position or velocity signals became unusable within several seconds. Therefore, an added step to solve this problem is needed. Here, a new method based on polynomial curve adjustment and splines approximation is proposed. In doing so, we will be able to achieve a correct Z-position overcoming the drift error problem.

Our correction method first tries to estimate the drift caused by a small DC bias in the Z-acceleration signal principally due to assuming a gravity component of 9.8 m/s2.This gross approximation leaves a small continuous component which gives rise to a quadratic component in the double-integrated signal. Here, a fourth order polynomial was used to obtain the estimation parameters from the position signal, without incurring in over-fitting. Then, the derivative of the estimated polynomial was employed to adjust the velocity signal and get the position signal through integration (Figure A4B).


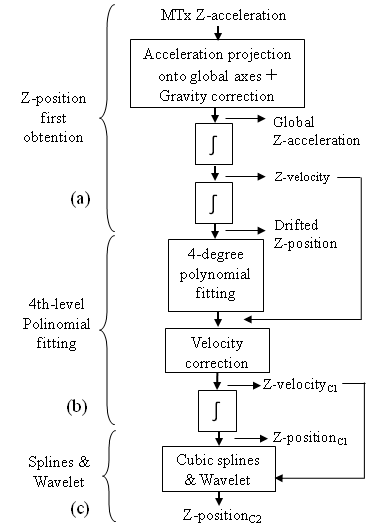


Figure A4. Z-position free-drift obtaining algorithm: double integration process, part (a), first correction (C1), part (b). and second correction (C2), part (c).

*Reference systems unification.*

Vicon reference system had to be changed to the global axes used by the MTx. To this purpose, some calibration measures from the Vicon system collected after each measurement were used to obtain the rotation matrix needed to make the coordinates change (Figure 2B)). This arrangement makes it possible to compare the trajectory reconstructed from ISU’s data and the one provided by the Vicon system.

***Statistical parameters for comparisons.***


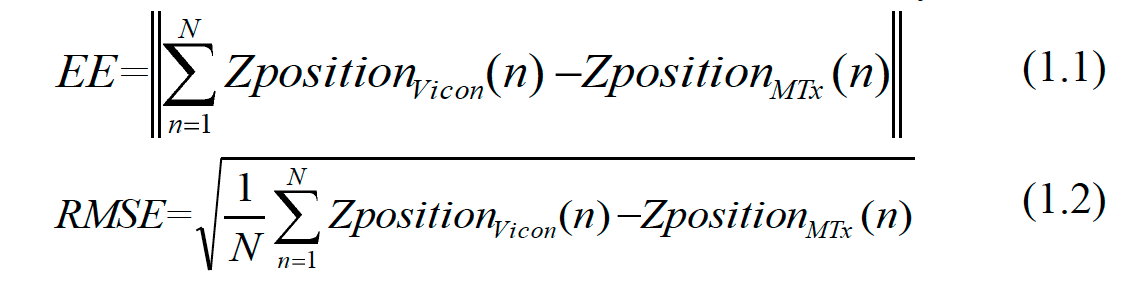
Comparisons were done based on parameters such as the Euclidean error (EE), (1.1), and accuracy, defined as the percentage of the whole signal without error. Furthermore, statistical parameters such as the root mean squared error (RMSE), (1.2), and the correlation coefficient (r) were also obtained to check our method's accuracy:

***Modified-BMFLC vs PB-algorithm***

The method reported in the present study was compared to a recent Modified-BMFLC drift-correction algorithm. The 30-s CST meets the quasi-periodic motion requirement for this drift-correction algorithm to be applied. In the literature there are other methods to correct the drift effect, but this was probably the first one which tried to cancel it when obtaining the position from the acceleration signal. Firstly, the cutoff frequency and the order of the high-pass filter were selected according to the 30-s CST conditions. A fourth level filter was chosen and the cutoff frequency was set at the movement’s fundamental frequency. Finally, in order to achieve a good BMFLC algorithm performance, 200 intermediate sub-frequencies were selected between the movement's fundamental and tenth harmonic frequencies.

Appendix B. UTHD manoeuvre phases breakdown in terms of centre of mass force orientation values. Descriptive statistics and effect size calculations.

| **Jumping Phase** | **Force Orientation in N (mean ± SD)** | **ACLR Injured Limb (n = 8)** | **ACLR Healthy Limb (n = 4)** | **Control Dominant Limb (n = 13)** | **Non-Dominant Limb (n = 14)** | **Significance (*p*)** | ***ES (difference)*** |
| --- | --- | --- | --- | --- | --- | --- | --- |
|  |  |  |  |  |  |  |  |
| **Propulsive Phase** | **X-axis** | 599.42 ± 155.98 | 607.15 ± 265.62 | 830.11 ± 365.95 | 507.22 ± 325.11 | *p* = 0.661 | *d = 0.82^* |
|  | 95% CI | 469.02 – 729.82 | 184.48 – 1029.81 | 597.59 – 1062.62 | 319.51 – 694.93 |  | *(large)* |
|  | **Y-axis** | 765.74 ± 222.55 | 891.21 ± 101.28 | 802.73 ± 203.81 | 797.74 ± 206.14 | *p* = 1.000 | *d = 0.173* |
|  | 95% CI | 579.68 - 951.79 | 730.05 – 1052.37 | 673.24 – 932.22 | 678.72 – 916.76 |  | *(small)* |
|  | **Z-axis** | 811.96 ± 137.67 | 790.16 ± 134.91 | 870.41 ± 182.6 | 915.11 ± 241.66 | *p* = 1.000 | *d = 0.361* |
|  | 95% CI | 696.87 – 927.06 | 575.49 – 1004.84 | 754.40 – 986.43 | 775.58 – 1054.65 |  | *(medium)* |
| **1^st^ Hop** | **X-axis** | 1293.57 ± 909.32 | 1202.87 ± 660.51 | 1451.71 ± 909.88 | 1498.56 ± 713.08 | *p* = 1.000 | *d = 0.174* |
|  | 95% CI | 533.36 – 2053.90 | 151.86 – 2253.9 | 873.60 – 2029.82 | 1086.84 – 1910.28 |  | *(small)* |
|  | **Y-axis** | 1037.29 ± 333.54 | 814.87 ± 257.09 | 802.2 ± 824.36 | 787.28 ± 704.69 | *p* = 1.000 | *d = 0.373* |
|  | 95% CI | 758.44 – 1316.13 | 405.78 – 1223.96 | 278.42 – 1325.97 | 380.4 – 1194.16 |  | *(medium)* |
|  | **Z-axis** | 2303.09 ± 797.26 | 2659.54 ± 477.15 | 2922.86 ± 778.01 | 2544.5 ± 666.32 | *p* = 0.404 | *d = 0.787* |
|  | 95% CI | 1636.57 – 2969.62 | 1900.29 – 3418.79 | 2428.54 – 3417.18 | 2159.78 – 2929.22 |  | *(large)* |
| **2^nd^ Hop** | **X-axis** | 1590.05 ± 957.49 | 1840.39 ± 611.25 | 1413.52 ± 942.99 | 1333.46 ± 942.99 | *p* = 1.000 | *d = 0.185* |
|  | 95% CI | 789.57 – 2390.53 | 867.75 – 2813.03 | 814.37 – 2012.66 | 1053.1 – 1613.83 |  | *(small)* |
|  | **Y-axis** | 868.44 ± 591.61 | 858.06 ± 193.06 | 493.76 ± 447.62 | 793.88 ± 563.89 | *p* = 0.707 | *d = 0.714* |
|  | 95% CI | 373.85 – 1363.04 | 550.86 – 1165.27 | 209.36 – 778.16 | 468.30 – 1119.46 |  | *(large)* |
|  | **Z-axis** | 2648.47 ± 857.21 | 2681.81 ± 847.16 | 3190.98 ± 915.12 | 2699.45 ± 674.86 | *p* = 0.915 | *d = 0.612* |
|  | 95% CI | 1931.82 – 3365.13 | 1333.79 – 4029.82 | 2609.54 – 3772.42 | 2309.80 – 3089.10 |  | *(medium)* |
| **3^rd^ Hop** | **X-axis** | 1969.65 ± 938.47 | 2071.64 ± 1504.19 | 2055.81 ± 1281.66 | 1838.99 ± 1017.65 | *p* = 1.000 | *d = 0.077* |
|  | 95% CI | 1185.07 – 2754.24 | 321.86 – 4465.14 | 1241.48 – 2870.14 | 1251.41 – 2426.57 |  | *(small)* |
|  | **Y-axis** | 1031.70 ± 875.99 | 1101.86 ± 592.34 | 336.1 ± 244.68 | 1091.64 ± 1324.03 | *p* = 0.315 | *d = 1.081* |
|  | 95% CI | 299.36 – 1764.05 | 159.31 – 2044.40 | 180.64 – 491.56 | 327.17 – 1856.11 |  | *(very large)* |
|  | **Z-axis** | 2773.44 ± 813.69 | 3468.91 ± 223.22 | 3444.95 ± 490.07 | 3277.19 ± 781.74 | *p* = 0.317 | *d = 0.999^* |
|  | 95% CI | 2093.18 – 3453.70 | 3113.71 – 3824.11 | 3133.58 – 3756.33 | 2825.82 – 3728.55 |  | *(very large)* |

Values are mean ± standard deviation, 95% confidence interval (inferior – superior value). P value from ANOVA calculations between ACLR injured limb and Control Dominant Limb. Standardised effect size interpreted as Cohen’s d values between ACLR injured limb and Control Dominant Limb. Abbreviations: UTHD, unilateral triple hop for distance; n, sample size; SD, standard deviation; 95% CI, 95% confidence interval; ES, effect size; *d*, Cohen’s *d*. * = *p* <.05. ^ = *d*> 0.8

Appendix C. UCOHD manoeuvre phases breakdown in terms of centre of mass force orientation values. Descriptive statistics and effect size calculations.

| **Jumping Phase** | **Force Orientation in N (mean ± SD)** | **ACLR Injured Limb (n = 8)** | **ACLR Healthy Limb (n = 4)** | **Control Dominant Limb (n = 13)** | **Non-Dominant Limb (n = 14)** | **Significance (p)** | ***ES (difference)*** |
| --- | --- | --- | --- | --- | --- | --- | --- |
|  |  |  |  |  |  |  |  |
| **Propulsive Phase** | **X-axis** | 469.97 ± 226.22 | 349.14 ± 238.81 | 297.88 ± 174.81 | 236.70 ± 196.74 | p = 0.049* | *d = 1.35^* |
|  | **95% CI** | 439.37 – 700.60 | 204.82 – 493.45 | 151.74 – 444.03 | 76.35 – 549.76 |  | *(very large)* |
|  | **Y-axis** | 731.12 ± 180.48 | 714.86 ± 102.75 | 647.14 ± 120.06 | 609.58 ± 64.196 | p = 1.000 | *d = 0.548* |
|  | **95% CI** | 626.91 – 835.32 | 652.77 – 776.95 | 546.79 – 747.51 | 507.43 – 711.73 |  | *(medium)* |
|  | **Z-axis** | 782.56 ± 233.48 | 745.28 ± 171.38 | 840.48 ± 419.5 | 629.25 ± 132.87 | p = 1.000 | *d = 0.170* |
|  | **95% CI** | 647.65 – 917.26 | 641.71 – 848.84 | 489.77 – 1191.19 | 417.83 – 840.68 |  | *(small)* |
| **1st Hop** | **X-axis** | 1220.44 ± 625.86 | 1067.51 ± 567.42 | 1161.92 ± 667.21 | 935.87 ± 324.24 | p = 1.000 | *d = 0.090* |
|  | **95% CI** | 859.08 – 1581.80 | 724.62 – 1410.39 | 604.11 – 1719.72 | 419.93 – 1451.81 |  | *(very small)* |
|  | **Y-axis** | 1183.73 ± 1205.41 | 982.58 ± 559.52 | 1149.43 ± 947.99 | 1820.19 ± 961.91 | p = 1.000 | *d = 0.032* |
|  | **95% CI** | 487.74 – 1879.71 | 644.47 – 1320.69 | 356.89 – 1941.98 | 289.57 – 3350.81 |  | *(trivial)* |
|  | **Z-axis** | 2684.76 ± 655.69 | 2545.92 ± 753.18 | 2307.59 ± 701.92 | 2627.54 ± 410.39 | p = 1.000 | *d = 0.555* |
|  | **95% CI** | 2306.24 – 3063.35 | 2090.78 – 3001.06 | 1720.77 – 2894.41 | 1974.51 – 3280.57 |  | *(medium)* |
| **2nd Hop** | **X-axis** | 2078.80 ± 1184.57 | 1307.29 ± 749.36 | 1899.84 ± 979.54 | 1263.94 ± 787.38 | p = 1.000 | *d = 0.165* |
|  | **95% CI** | 1394.85 – 2762.76 | 854.46 – 1760.12 | 1080.92 – 2718.75 | 11.04 – 2516.83 |  | *(small)* |
|  | **Y-axis** | 1287.44 ± 990.76 | 887.71 ± 558.86 | 1315.97 ± 559.21 | 1744.57 ± 435.83 | p = 1.000 | *d = 0.035* |
|  | **95% CI** | 715.39 – 1859.49 | 549.99 – 1225.42 | 848.46 – 1783.48 | 1051.07 – 2438.08 |  | *(trivial)* |
|  | **Z-axis** | 2697.85 ± 671.88 | 2434.93 ± 919.48 | 2276.73 ± 470.11 | 2239.18 ± 1128.61 | p = 1.000 | *d = 0.726* |
|  | **95% CI** | 2309.92 – 3085.78 | 1879.32 – 2990.55 | 1883.71 – 2669.74 | 443.32 – 4035.05 |  | *(large)* |
| **3rd Hop** | **X-axis** | 1611.41 ± 1017.16 | 1753.74 ± 551.85 | 1729.41 ± 977.62 | 1245.67 ± 294.01 | p = 1.000 | *d = 0.118* |
|  | **95% CI** | 1024.12 – 2198.70 | 1420.26 – 2087.22 | 912.10 – 2546.71 | 777.83 – 1713.50 |  | *(small)* |
|  | **Y-axis** | 921.57 ± 890.30 | 759.77 ± 591.66 | 1321.52 ± 709.56 | 543.63 ± 178.89 | p = 1.000 | *d = 0.496* |
|  | **95% CI** | 407.53 – 1435.62 | 402.23 – 1117.30 | 728.31 - 1973 | 258.98 – 828.28 |  | *(medium)* |
|  | **Z-axis** | 2873.45 ± 540.75 | 3022.63 ± 698.38 | 2306.72 ± 724.34 | 2628.47 ± 745.60 | p = 0.353 | *d = 0.887^* |
|  | **95% CI** | 2561.23 – 3185.68 | 2600.61 – 3444.66 | 1701.16 – 2912.28 | 1442.05 – 3814.88 |  | *(large)* |

Values are mean ± standard deviation, 95% confidence interval (inferior – superior value). P value from ANOVA calculations between ACLR injured limb and Control Dominant Limb. Standardised effect size interpreted as Cohen’s d values between ACLR injured limb and Control Dominant Limb. Abbreviations: UTHD, unilateral triple hop for distance; n, sample size; SD, standard deviation; 95% CI, 95% confidence interval; ES, effect size; *d*, Cohen’s *d*. * = *p* <.05. ^ = *d*> 0.8.
